# A large-scale dataset for mitotic figure assessment on whole slide images of canine cutaneous mast cell tumor

**DOI:** 10.1038/s41597-019-0290-4

**Published:** 2019-11-21

**Authors:** Christof A. Bertram, Marc Aubreville, Christian Marzahl, Andreas Maier, Robert Klopfleisch

**Affiliations:** 1 Institute of Veterinary Pathology, Freie Universität Berlin, Berlin, Germany; 2 Pattern Recognition Lab, Computer Science, Friedrich-Alexander-Universität Erlangen-Nürnberg, Erlangen, Germany

**Keywords:** Mitosis, Cancer

## Abstract

We introduce a novel, large-scale dataset for microscopy cell annotations. The dataset includes 32 whole slide images (WSI) of canine cutaneous mast cell tumors, selected to include both low grade cases as well as high grade cases. The slides have been completely annotated for mitotic figures and we provide secondary annotations for neoplastic mast cells, inflammatory granulocytes, and mitotic figure look-alikes. Additionally to a blinded two-expert manual annotation with consensus, we provide an algorithm-aided dataset, where potentially missed mitotic figures were detected by a deep neural network and subsequently assessed by two human experts. We included 262,481 annotations in total, out of which 44,880 represent mitotic figures. For algorithmic validation, we used a customized RetinaNet approach, followed by a cell classification network. We find F1-Scores of 0.786 and 0.820 for the manually labelled and the algorithm-aided dataset, respectively. The dataset provides, for the first time, WSIs completely annotated for mitotic figures and thus enables assessment of mitosis detection algorithms on complete WSIs as well as region of interest detection algorithms.

## Abstract (summary)

**Table:** 

Measurement(s)	Mitotic Figure
Technology Type(s)	visual observation method • machine learning
Factor Type(s)	tumor grade • experimental method
Sample Characteristic - Organism	Canis

Machine-accessible metadata file describing the reported data: 10.6084/m9.figshare.9952469

## Background & Summary

Microscopy image recognition has seen vast advances in recent years, fostered by the availability of high quality datasets as well as by the application of sophisticated deep learning pipelines. One of the most important topics in the field of microscopy imaging is the classification of cells, typically stained with hematoxylin and eosin (H&E) dye. In this area, one particularly challenging task is the detection of mitotic figures, i.e. cells undergoing division, in tumor tissue. It is commonly accepted that the quantity of mitotic figures is one of the most powerful prognosticators of biological behavior for many tumor types, both in humans^1,2^ and animals^3–5^. In the field of automatic detection of those mitotic figures, there have been a number of competitions in recent years, e.g. the TUPAC16 challenge^6^, the ICPR MITOS-2012^7^ and ICPR MITOS-ATYPIA-2014 challenge^8^.

Mitotic figures are defined histologically by the lack of a nuclear membrane and the presence of hairy projections of the chromosomes (nuclear material)^9^. A common method for quantification is the mitotic count (MC), which means counting mitotic figures in a standard-sized area located where the tumor is assumed to have the highest mitotic density. The method is widely used, as it can be obtained easily on standard H&E stained sections without additional costs^10^. Regardless, reproducibility is currently hampered by high inter- and intra-rater variability^11,12^ due to the difficulty of identifying mitotic figures and the variable distribution of mitotic figures throughout the tumor section^13^. Identification of individual mitotic figures has only moderate agreement between trained pathologists as they include a wide range of morphological variants depending on the phase of cell division and tissue properties as well as atypical morphologies. Previous studies have identified inter-rater disagreement of 17.0–34.0% in distinguishing individual mitotic figures from other cell structures in canine mast cell tumors (CCMCT) and human breast cancer^12–14^. Yet, even if results are typically more stable, algorithmic approaches have not reached human performance in this task. Identifying the area with the highest mitotic density – as requested for the MC – is complicated by a patchy mitotic distribution^13^. In contrast to human observers, machine learning-based algorithms can quickly evaluate entire whole slide images (WSI) and propose the area with the highest density. A previous study has shown that algorithms can outperform human observers in this task and pose a very promising method to overcome this challenge^15^.

CCMCT are among the most common skin tumors in dogs^16^. Tumors compose of round to polygonal neoplastic mast cells with variable amounts of faintly stained intracytoplasmic granules, which contain different substances such as eosinophilic chemotactic factors. Due to these substances, aggregation of non-neoplastic eosinophilic granulocytes – a small immune cell containing eosinophilic granules – is additionally found in most CCMCT^17^. Biological behavior is highly variable: CCMCT are considered potentially malignant. Whereas the majority of cases will have a benign behavior, others may develop fatal metastatic diseases. Therefore, accurate prognostication of the biological behavior such as by quantification of mitotic figures is essential in order to select an appropriate therapeutic approach^16^. It has been determined that the MC has good prognostic value for CCMCT as a solitary parameter^3,4^ and as part of a grading system^18^.

Given the importance of quantifying mitotic figures in various tumor types of animals and humans, it is at first glance surprising that none of the available datasets provide labels for complete WSI. Manual annotation of such large areas, however, is a labor-intense and tedious task. In this work, we present a dataset consisting of 32 fully-annotated WSI of CCMCT with a total of 44,880 mitotic figure annotations. Potential mitotic figures have been identified by one veterinary pathologist [CB] and subsequently by a deep learning-based pipeline. Two experts [CB, RK] classified the annotations in a blinded manner and reviewed the disagreed labels to find common consensus on the label class. This collection^19^ represents the currently largest data set in number of annotated mitotic figures and annotated tumor area. Therefore it provides researchers with new opportunities for the development and refinement of data-driven algorithms for mitotic figure identification on entire whole slide images.

## Methods

### Selection and preparation of specimen

Histological specimens of CCMCT cases were obtained from the author’s institute diagnostic archive. 32 Cases with high tissue quality were selected retrospectively in such a way that the dataset includes cases with variable density of mitotic figures ranging from low to very high MCs. One representative tissue block (formalin-fixed and paraffin-embedded) was chosen per case. New tissue sections were produced at a thickness of approximately 1 *μm* and stained with H&E by a tissue stainer (ST5010 Autostainer XL, Leica, Germany). Whole slide scanning was performed by a linear scanner (ScanScope CS2, Leica, Germany) in one focal plane by default settings at a magnification of 400x (image resolution: 0.25 *μm*/pixel), using an Olympus UPlanSAPO 20x lens (field number = 26.5, numerical aperture = 0.75).

### Manually expert labelled (MEL) dataset

**Fig. 1:**
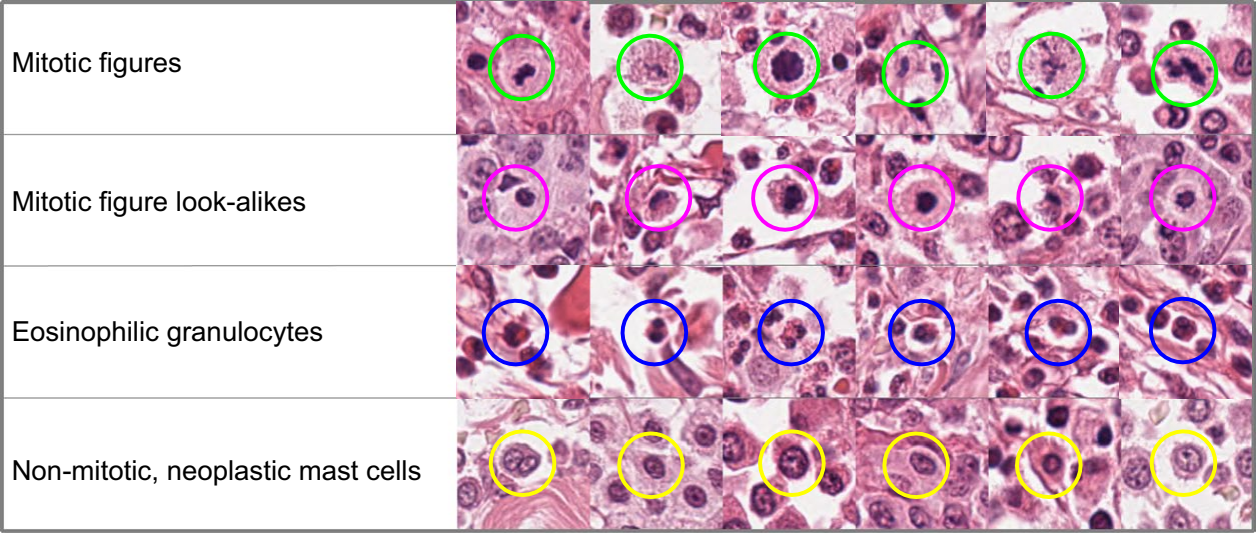
Examples for various cell types annotated in the data set. Not shown are ambiguous cells. Due to their count, only for the class of mitotic figures a complete list of cells is provided.

**Fig. 2:**
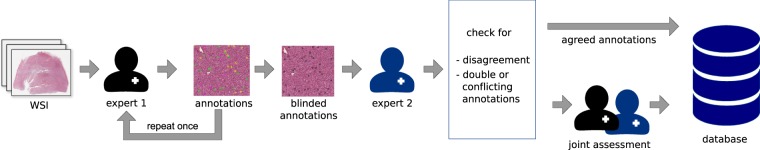
Creation of the manually expert labelled (MEL) dataset variant, which is the base for all other data set variants. Every WSI was screened for mitotic figures by the first expert. The second expert was able to see annotations but not class labels, and was additionally able to set new annotations, if needed. Disagreed cells were re-assigned to both experts for a common consensus.

Primary annotations were carried out by two experts trained in the field of veterinary pathology [CB, RK]. For this, we used an open source software solution made available by our research group^20^. This software provides two modes specifically designed for this task: Firstly, an expert can screen a WSI for specific structures (in this case mitotic cells) at highest magnification. For this, the software detects tissue presence in the image and shows partially overlapping tissue segments to the expert for assessment. This ensures that no region of the WSI is left out for assessment. The first expert on each dataset classified cells into the following groups (see also Fig. 1):

1. Mitotic figure.
2. Non-mitotic, neoplastic mast cells.
3. Non-mitotic, ambiguous cells.
4. Eosinophilic granulocytes.

The group of *ambiguous cells* plays a special role, here, as it is non-disjunct to the other groups besides *mitotic cell*. This group was initially used to account for cells that are not mitotic figures, but also not clearly attributed to other cells.

The first assessment of a WSI was always carried out twice by the first expert (see Fig. 2). The second expert was blinded to the cell class decisions of the first expert, but not to the positions where cells were annotated. We followed this procedure, because we assumed the risk to miss rare mitotic events on WSIs to be greater than the potential bias introduced when having to judge an already available cell annotation of unknown class. The annotation software^20^ provides a mode for this blinded annotation, in which one or multiple unassigned annotations are presented without any class labels. After selection of the respective classes, the next random annotation(s) would be presented.

It is well known, that the concordance of different experts w.r.t. mitotic figure assessment is not perfect. All cases, where both experts did not agree on the same class, and additionally a number of doubtful candidates found by the first reviewer, were re-evaluated by both experts in order to find agreement on the label class, resulting in the *manually expert labelled* (MEL) data set variant. Naturally, manual screening of large images introduces the risk of missing candidates for annotation, which we perceive as one of the main risks for data quality. Due to this, we employed an algorithm-aided pipeline.

### Augmented dataset for mitotic figures

In order to improve the quality of our dataset, we made use of deep learning techniques, trained on the manually, expert-labelled (**MEL**) data set. We derive two data set variants:

#### Hard-example augmented expert labelled dataset variant (HEAEL)

In this dataset variant, our primary aim was to split up the group of non-mitotic figures and ambiguous cells into mitotic figure-lookalikes and other cells. It has been shown that determination of hard examples is helpful for faster convergence of the classification approaches^21^.

**Fig. 3:**
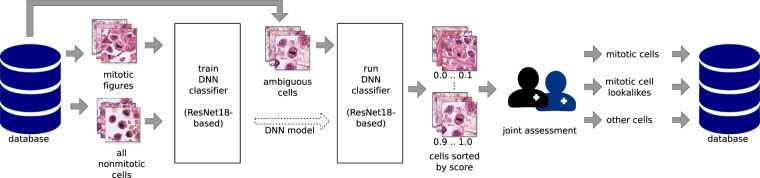
Algorithm-aided division of the ambiguous class *non-mitotic cells*, resulting in the hard-example augmented expert labelled (HEAEL) dataset variant. A ResNet18^22^-based classifier was used to sort ambiguous cells into more or less likely mitotic figure candidates, which were subsequently presented to both experts.

For cell classification, we used a standard CNN network architecture based on ResNet-18^22^ as backbone. We trained this network using image crops of 128 px × 128 px around annotated cells of the dataset. The cases where this cell classifier network predicted a high certainty mitotic figure were reviewed again by both experts, to account for potentially misclassified cells (see Fig. 3).

#### Object-detection augmented expert labelled dataset variant (ODAEL)

**Fig. 4:**
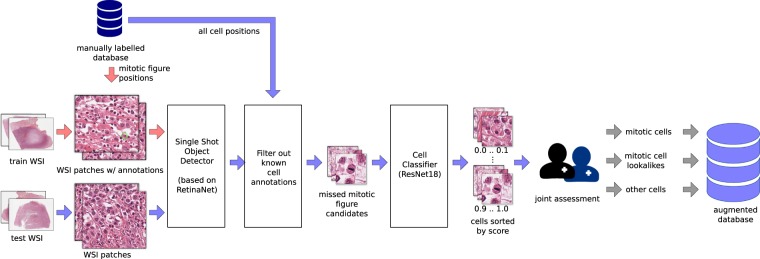
Algorithm-aided labelling of potentially missed mitotic cells, resulting in the object-detection augmented expert labelled (ODAEL) dataset variant. We used a customized RetinaNet^23^ object detector for mitotic figure candidate extraction from WSI, subsequently filtered out known cells and performed a refining classification. Results of which were presented to two experts to extend the database with potentially missed mitotic figures.

In order to counteract bias encountered due to one or both experts missing candidates of the (relatively rare) mitotic figures, we shifted towards an augmented dataset generation technique. In this approach, a deep network would propose additional potential mitotic figure candidates, and the human experts would have to rate and assign to the different groups of our dataset (see Fig. 4). With this mechanism, we generated, additionally to the missed mitotic figures, also a list of hard negative samples, i.e. examples that a model or even a human expert could potentially misjudge for true mitotic figures. By definition, hard negative mitotic figure lookalikes were cells where the model classified a mitotic figure, but the consensus of human experts neglected this to be the correct label.

First, based on a three-fold split, a custom RetinaNet^23^ model was trained for each fold. We used an input size of 512 × 512 for the model, and fed images that would typically contain at least one mitotic figure to the model. RetinaNet uses focal loss to account for class imbalance, which is especially important in our case due to the foreground (mitotic figure) class being less prevalent than the background class. As network backbone, we used a ResNet-18^22^ topology, pre-trained on ImageNet^24^. We trained the model for 6 cycles, each with 50,000 random image crops.

Li *et al*.^25^ have shown that a dual stage approach improves performance significantly over a single stage object detection approach. Motivated by this, we introduce a second stage cell classifier after the initial object detection/cell localization stage. We use the previously trained (for hard-example classification) network for this purpose.

## Data Records

We provide the 32 original WSI in the Aperio SVS format on *figshare*^19^. All slides have been fully anonymized and label images have been removed. Each described variant of the dataset is made available as database file (SQLite3 format). The database format provides for each annotation:

- The slide on which the cell was annotated.
- The coordinates (x, y) of the cell.
- The agreed class (by all experts) of the cell.
- Two or more individual class labels. For each label, it is known who assigned the label, be it expert 1, expert 2, both experts (consensus vote), or, for the augmented dataset the object detection algorithm. The unique numeric identifier of each label also represents the order in which the labels were given to the annotation.

Table 1 gives an overview about all three dataset variants. Slides are sorted by number of annotated mitotic figures. There was a large spread in the total count, reflecting also differences in tumor proliferation. To ease comparison of results on the dataset, we assigned slides randomly to be part of the training or test dataset. The number of *mitotic figure look-alikes* greatly increased from the hard-example-augmented dataset to the object-detection-augmented dataset. The reason for this is that all non-mitotic cells that were given a probability of above 0.5 for mitosis by the dual stage classifier were added to this class.

**Table 1:** Overview of the dataset and all its variants: Numbers given per cell category are for the variant where expert labels were given after object detection/hard example classification/only manual observation.

Slide name	Tumor area	Mitotic figures	Mitotic figure look-alikes	Granulocytes	Normal tumor cells set	Set
2f2591b840e83a4b4358.svs	144.79 mm^2^	3/1/1	48/2/0	2213/2213/2213	1149/1113/1113	train
ce949341ba99845813ac.svs	13.94 mm^2^	4/1/1	30/2/0	35/35/35	1200/1197/1197	train
91a8e57ea1f9cb0aeb63.svs	25.24 mm^2^	6/2/2	16/3/0	573/573/573	1916/1903/1903	train
9374efe6ac06388cc877.svs	35.63 mm^2^	7/6/6	17/4/0	1531/1531/1531	1567/1534/1534	train
0e56fd11a762be0983f0.svs	25.63 mm^2^	8/4/4	262/17/0	239/239/239	1620/1089/1089	train
dd6dd0d54b81ebc59c77.svs	62.25 mm^2^	11/5/5	57/15/0	1230/1230/1230	1830/1733/1733	train
be10fa37ad6e88e1f406.svs	14.87 mm^2^	12/3/3	55/2/0	124/124/124	1354/1351/1351	test
2e611073cff18d503cea.svs	81.64 mm^2^	18/11/11	137/2/0	2556/2556/2556	1136/1111/1111	train
066c94c4c161224077a9.svs	115.49 mm^2^	19/19/19	54/10/0	1742/1742/1742	1035/1001/1001	train
285f74bb6be025a676b6.svs	83.07 mm^2^	19/14/14	48/4/0	2895/2895/2895	1837/1807/1807	train
f3741e764d39ccc4d114.svs	39.23 mm^2^	37/28/28	115/9/0	724/724/724	1932/1903/1903	test
c86cd41f96331adf3856.svs	189.02 mm^2^	56/39/39	75/2/0	2412/2412/2412	1593/1548/1548	test
2efb541724b5c017c503.svs	21.27 mm^2^	66/66/66	24/14/0	645/645/645	621/557/557	train
70ed18cd5f806cf396f0.svs	88.30 mm^2^	85/68/4	880/267/0	1913/1913/1913	578/543/543	train
552c51bfb88fd3e65ffe.svs	185.63 mm^2^	119/68/68	670/9/0	1688/1688/1688	2074/2050/2050	test
3f2e034c75840cb901e6.svs	103.25 mm^2^	571/547/543	350/79/0	1434/1434/1434	1913/1547/1547	train
8c9f9618fcaca747b7c3.svs	312.96 mm^2^	715/675/675	1212/546/0	28/28/28	3077/2974/2974	test
c91a842257ed2add5134.svs	160.25 mm^2^	759/716/716	690/128/0	2327/2327/2327	1719/1584/1584	test
dd4246ab756f6479c841.svs	238.22 mm^2^	777/731/729	525/84/0	2703/2703/2703	2986/1917/1917	test
8bebdd1f04140ed89426.svs	213.66 mm^2^	1000/976/958	534/276/0	1563/1563/1563	2196/1791/1791	train
2f17d43b3f9e7dacf24c.svs	87.86 mm^2^	1157/1097/1097	477/49/0	2719/2719/2719	1625/1593/1593	train
a0c8b612fe0655eab3ce.svs	261.85 mm^2^	1279/1210/1210	1407/110/0	2118/2118/2118	1556/1522/1522	train
ac1168b2c893d2acad38.svs	346.26 mm^2^	1329/1316/1310	474/288/0	613/613/613	4354/2427/2427	train
fff27b79894fe0157b08.svs	256.29 mm^2^	1744/1545/1544	1466/166/0	5774/5774/5774	2279/1805/1805	train
34eb28ce68c1106b2bac.svs	190.18 mm^2^	2279/1879/1878	1297/47/0	2054/2054/2054	1540/1532/1532	train
f26e9fcef24609b988be.svs	136.58 mm^2^	2380/2341/2341	459/168/0	2447/2447/2447	1807/1655/1655	test
96274538c93980aad8d6.svs	188.35 mm^2^	3068/2978/2975	3762/733/0	1170/1170/1170	4297/1703/1703	test
add0a9bbc53d1d9bac4c.svs	242.71 mm^2^	3569/3393/3387	1759/477/0	415/415/414	2198/1977/1977	test
39ecf7f94ed96824405d.svs	220.56 mm^2^	3689/3516/3508	3412/767/0	1572/1572/1572	1931/1678/1678	train
20c0753af38303691b27.svs	269.48 mm^2^	4343/4048/4037	2024/343/0	1772/1772/1772	3835/1668/1668	train
c3eb4b8382b470dd63a9.svs	149.74 mm^2^	4767/4705/4696	1326/564/0	140/140/140	9461/9383/9383	train
1018715d369dd0df2fc0.svs	337.88 mm^2^	10984/10599/10590	4303/912/0	2070/2070/2070	3135/2137/2137	test

Not shown in this table are ambiguous cells.

### Getting started

To reconstruct the experiments, the first step is to clone the GitHub repository (an overview is given in Table 2). It includes a jupyter notebook (Setup.ipynb) that downloads all individual slides and the database file from *figshare*. After this initial setup was run, all required data is available to run the other notebooks. Training of the networks is conducted in the notebooks RetinaNet-CCMCT-<variant>.ipynb, where <variant> is one of the data set variants (MEL, HEAEL, ODAEL). Trained networks are stored as RetinaNet-<variant>-export.pth in the main folder. Also in the main folder, there is a script to run the models on the test set (Inference-Retinanet.py) and the evaluation scripts to calculate the F1 score. In the subfolder 2nd_stage, all scripts and notebooks are provided to train and evaluate the 2nd stage ResNet-18 classifier. First, patches need to be extracted (exportDataset_<variant>.py), and later the classifier is trained (CellClassification-<variant>.ipynb). For inference, there is a third script (Inference-CellClassifier.py) available. Evaluation of both stages and all variants is performed in the notebook Evaluation.ipynb in the root folder.

**Table 2:** Excerpt from the GitHub file list.

Root folder	Description
RetinaNet-CCMCT-ODAEL.ipynb	Training of RetinaNet on the ODAEL data set variant.
Inference-RetinaNet.py	Inference script to test all RetinaNet models.
Evaluation.ipynb	Evaluation notebook for all RetinaNet models (1^st^ and 2^nd^ stage).
AblationStudy_Evaluation.ipynb	Evaluation of the ablation study.
Setup.ipynb	Download of all databases and WSIs from figshare.
**Folder** 2nd_stage	**Description**
CellClassification-ODAEL.ipynb	Training of a 2nd stage cell classifier on the ODAEL data set variant
Inference-CellClassifier.py	Inference script to test the 2nd stage classifier on results of the 1st stage
exportDataset_ODAEL.py	Script to export image patches of the ODAEL data set (needed for classifier training)

Only main files are being discussed, and only the ODAEL data set variant, however all results discussed in this work are available in the repository.

## Technical Validation

Our technical validation of the dataset is two-fold: First, we assessed the quality of assigned labels by conducting a classification experiment of mitotic figures versus other cells. Secondly, we performed a detection task on the complete WSIs of the test set. Both are informative for distinctive questions: While the first test can yield information as to how well separation of classes is possible and thus indirectly assesses label class quality, the latter also assesses the coverage of mitotic figures on the WSI.

### Classification of preselected cells

For this validation task, 128 × 128 px patches with single cells of all classes besides ambiguous cells at their respective center (mitotic figure, mitotic figure lookalike, neoplastic mast cells and granulocyte) have been extracted from the ODAEL variant of the dataset. We used a standard state-of-the-art classification CNN classification network, based on a ResNet-18 stem^22^ pre-trained on ImageNet^24^. The network was trained for 1 cycle of 10 epochs using the super-convergence scheme^26^ with a maximum learning rate of 10^−2^ and the Adam optimizer^27^. With this approach, we reach an accuracy of 91.390% on the test set. As shown in Table 3, the main confusion is between mitotic figures and mitotic figure-lookalikes, while all other cell types were separated well by the classifier. This result also is consistent with the high intra- and inter-rater variance in this task by human experts.

**Table 3:** Confusion matrix: Classification results of a ResNet-18-based CNN classifier on patches with a certain cell type in the center (accuracy on test set is 91.390%).

Actual	pred. mitotic fig.	pred. mitotic fig. look-alike	pred. granulocyte	pred. tumor cell
Mitotic figure	19478	2985	10	3
Mitotic figure look-alike	2942	10582	57	44
Granulocyte	1	66	16011	30
Tumor cell	3	92	53	20651

### Detection of mitotic figures on WSI

This task was performed to give a baseline for mitotic figure detection on our dataset. We trained one model for each of the dataset variants. For this, we chose RetinaNet^23^ as a state-of-the-art object detection approach, because implementations are available for all major machine learning frameworks currently in use in the scientific community. A similar approach was also followed by Li *et al*. in their DeepMitosis framework^25^. RetinaNet introduced the focal loss, which is very suitable for mitotic figure detection, because it assigns greater weight to decisions that were hard for the network, and thus an explicit hard example mining as a training strategy can be avoided.

We feed 256 × 256 px image patches to our model, which is build on a ResNet-18^22^ stem pre-trained on ImageNet^24^ with spatial pyramid features for the network, and two customized heads, one for bounding box detection and one for mitotic figure/background classification. The heads are based on the lowest feature pyramid layer at the highest (16 × 16) spatial resolution.

We used a customized sampling scheme to ensure and speed up model convergence. For each training batch, 50% of the images would contain at least one mitotic figure, 40% would contain a mitotic figure look-alike (hard example) and 10% of images were picked completely at random from the slide. In the MEL dataset variant where no hard examples were available, we used the ambiguous cells instead in the scheme. For training, only the upper half of each WSI was used, for validation, we used the lower half. The test set was never used during training and algorithmic optimization.

Due to the high number of potential images to be extracted from the WSI, we perceive the classical definition of epochs in deep learning (i.e. the entire training set being seen in back-propagation at least once) to be not sensible any more. We thus consider pseudo-epochs of 5,000 (each time randomly selected) images for our training.

After initial training for a single pseudo-epoch, the heads of the networks were trained using the super-convergence scheme of Smith and Topin^26^ with Adam as optimizer^27^ for 3 cycles of 10 pseudo-epochs using a maximum learning rate of 10^−4^. After this convergence, the complete network was fine-tuned for 2 × 30 pseudo-epochs for which an early stopping paradigm was applied to retrieve the model with highest validation performance. As per the validation loss, we did not find the model to overfit the data, which is not surprising due to the huge amount of image material in the data set. The sampling scheme used by us leads to an overestimation of likelihood for mitotic figures by the model. Due to this, we optimize the threshold for object detection by processing the complete WSIs of the training and validation set after the model was trained. Again, we used the patch classifier trained in the previous step as second stage for the mitotic figure detection.

Not surprisingly, we find an influence of the dataset variant on the F1 score (see Table 4). Since the ODAEL variant is expected to be thorough in the identification of all present mitotic figures, it is in line with expectations that the ODAEL variant archived the highest F1 scores for all models. Overall, the influence of the dataset variant on the F1 score is above 3 percentage points, underlining the sensibility of the applied method.

**Table 4:** Performance assessment (F1 score) on the different variants of the dataset.

Model	RetinaNet			RetinaNet + 2nd stage (ResNet-18)		
	MEL as test	HEAEL as test	ODAEL as test	MEL as test	HEAEL as test	ODAEL as test
MEL as train	0.610	0.607	0.616	0.786	0.786	0.795
HEAEL as train	0.615	0.615	0.625	0.755	0.755	0.764
ODAEL as train	0.620	0.620	**0**.**628**	0.810	0.810	**0**.**820**

### Ablation study

One of the most interesting questions for a dataset of this size is, how strongly it benefits from the increased size over previous approaches. The predominant approach in current datasets is to annotate a subset of a size of ten contiguous high power fields (HPF). We follow the definition of Meuten^10^, who defined the area of a single HPF to be 0.237 mm^2^. To investigate, how a restriction in size impacts the detection results, we thus derived small subsets with an area of 5, 10 and 50 HPF, taken from our best performing ODAEL dataset variant. We asked a senior pathology expert to determine the most mitotically active part of the tumor as he would do for manual mitotic counts. This procedure is consistent with the one described by Veta *et al*. for the TUPAC16 dataset^6^.

**Fig. 5:**
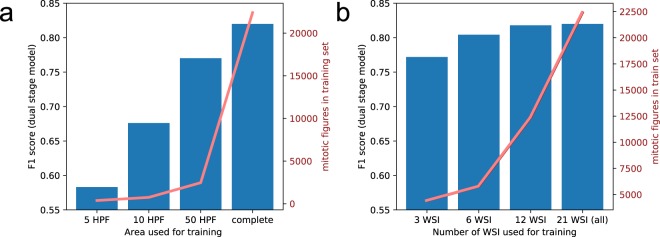
Results of the ablation study using the dual stage detector. In panel a, the results of using varying training area sizes around an expert-selected most mitotically active part of tumor are given. In panel b we show the results of using only a subset of the slides for training.

To compare against the existing data sets, we focus in the following on the data set reduced to 10 HPF area (see Table 5 for the other cases). Using an aspect ratio of 4:3, the resulting images were 7,017 px in width and 5263 px in height. The resulting (to an area of 10 HPF reduced) dataset consists of 7,617 cell annotations, including 1041 mitotic figures. Regardless having a slightly higher number of cases, it includes a quite similar number of mitotic figures than the AMIDA13 dataset (cf. Table 6). We trained the same pipeline as for the complete dataset, however for a shorter amount of iterations to avoid over-fitting due to the much smaller dataset variance: The RetinaNet object detector was trained for a single cycle of 10 pseudo-epochs using super-convergence, and for another 60 iterations with normal adaptive learning rate based on Adam. During this last period, we used early stopping and chose the model with highest validation performance. As shown in Fig. 5, the performance of the model increases significantly with the amount of annotated area and the number of available WSI. The data shows, however, that a plateau is reached for the number of WSI, and doubling the number of training WSI from 12 to all (21) increased performance only slightly.

**Table 5:** Ablation study dataset subsets.

Area covered	Image size	Mitotic figures	Mitotic figure lookalikes	Total cell annotations
5 HPF	4962 × 3721	526	219	3,497
10 HPF	7017 × 5263	1,041	435	7,617
50 HPF	15692 × 11769	3,916	1,850	27,177

Areas have been selected as areas around the center of a 10 HPF spanning reference area selected by a senior pathology expert as area with highest mitotic activity.

**Table 6:** Comparison of our dataset and its variants to other datasets with mitotic figure annotations.

Dataset	Annotations	Mitotic figures	Mitotic figure look-alikes	Tumor cases	Annotated tumor area
MITOS 2012^7^	226	226	0	5	13.11 mm^2^
MICCAI AMIDA 13^29^	1,083	1,083	0	23	151.500 mm^2^
MITOS-ATYPIA 2014 (training set)^8^	3,633	749	2,884	11	153.16 mm^2^
TUPAC 2016 (training set)^6^	1,552	1,552	0	73	251.500 mm^2^
MITOS_WSI_CCMCT_MEL	238,340	42,465	0	32	4,842.062 mm^2^
MITOS_WSI_CCMCT_HEAEL	238,339	42,607	6,099	32	4,842.062 mm^2^
MITOS_WSI_CCMCT_ODAEL	**262**,**481**	**44**,**880**	**27**,**965**	32	4,842.062 mm^2^

For the more recent datasets MITOS 2014 and TUPAC2016, only training sets are available. AMIDA13 is no longer available, but is part of the TUPAC16 dataset.

## Usage Notes

Annotations are provided in the SlideRunner database format^20^, which can be also used to view the WSIs with all annotations, but also in the popular MS COCO format. Be aware that the latter does not provide the possibility to annotate an object with multiple expert labels, thus the data format is of reduced information content. We encourage to view and process the data based on the SlideRunner database format.

## Data Availability

All code used in the experiments described in the manuscript was written in Python 3 and is available through our GitHub repository (https://github.com/maubreville/MITOS_WSI_CCMCT/). We provide all necessary libraries as well as Jupyter Notebooks allowing tracing of our results. The code is based on fast.ai and OpenSlide^28^ and provides some custom data loaders for use of the dataset.
